# A Generative AI–Based Technical Data Extraction Tool for IoT Application Systems

**DOI:** 10.3390/s26041081

**Published:** 2026-02-07

**Authors:** Dezheng Kong, Nobuo Funabiki, Htoo Htoo Sandi Kyaw, I Nyoman Darma Kotama, Zihao Zhu, Alfiandi Aulia Rahmadani

**Affiliations:** Department of Information and Communication Systems, Okayama University, Okayama 700-8530, Japan; pxep6n2q@s.okayama-u.ac.jp (D.K.); htoohtoo@okayama-u.ac.jp (H.H.S.K.); pjco5fyn@s.okayama-u.ac.jp (Z.Z.);

**Keywords:** internet of things, AI, retrieval-augmented generation, vector database, schema-based extraction, data sheet, technical information

## Abstract

Nowadays, *Internet of Things (IoT)* application systems play an essential role in smart cities, industry, healthcare, agriculture, and smart homes. For non-expert users, designing and implementing IoT application systems remains challenging, especially when configuring sensors, edge devices, and server platforms. To support configuration tasks of IoT application systems, we have developed an *AI-based setup assistance tool*. However, AI models still fail to reliably support newly released or previously unseen devices, sometimes producing incomplete or erroneous outputs that may lead to configuration failures. Incorporating their technical-document information into *Retrieval-Augmented Generation (RAG)* is an effective way to supplement AI knowledge and improve reliability. In this paper, we propose a *generative AI-based technical data extraction tool* to address the challenges. It extracts essential technical information using the *schema-based extraction* from given PDF or HTML datasheets and converts it into a structured format suitable for AI-supported configurations. A local *vector database* is used to enable semantic similarity retrieval and provide document-grounded evidence for *RAG*-based answering, ensuring consistent support for previously unseen IoT devices. For evaluations, we applied the proposal to several sensor and device datasheets and compared extracted specifications with ground-truth values to measure accuracy and completeness. Then, we compared end-to-end configuration QA reliability against a commercial baseline (*ChatPDF*) using the golden benchmark. The results show that the proposed tool reliably acquires key specifications and significantly improves end-to-end configuration QA reliability. Across 960 golden QA pairs, the proposed method improves *Recall* from 0.636 to 0.926 and *Accuracy* from 0.595 to 0.807 compared with *ChatPDF*.

## 1. Introduction

**Figure 1:**
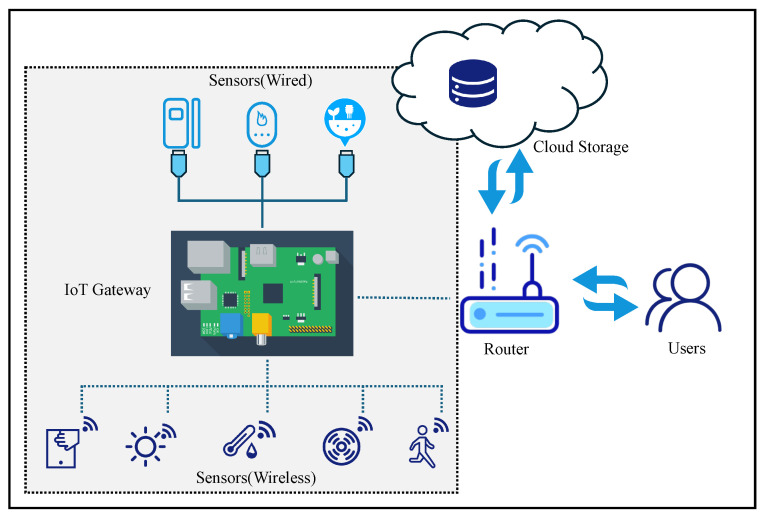
Example IoT application system.

The rapid expansion of *Internet of Things* (IoT) application systems has enabled their large-scale deployment in smart cities [1,2], industrial automation, healthcare, agriculture, and personal-use environments [3,4,5]. Then, as IoT devices continue to diversify, users must combine heterogeneous sensors, edge devices, and server platforms to build functional systems [6]. However, non-expert users often struggle with system configurations due to varying interfaces, communication protocols, and device-specific requirements. As shown in Figure 1, a locally deployed IoT application system comprises multiple functional layers, including wired and wireless sensors, an IoT gateway, a router, and cloud storage services. Among these components, the heterogeneity of sensor types and connection modalities remains the most challenging aspect, particularly for inexperienced users, as it requires precise interpretations of device-specific interfaces and operational constraints.

In standardized environments such as consumer smart home appliances and secure storage systems, device self-identification is often handled via machine-readable formats like *XML*-based Device Descriptors or plug-and-play protocols [7,8]. These methods allow for highly efficient configuration with minimal computational overhead. However, unlike these specific domains, the broader market of component-level IoT sensors and legacy industrial hardware typically lacks the onboard storage to host description files. Instead, their specifications are often locked in unstructured PDF datasheets. Therefore, our proposed tool, which is capable of automatically extracting structured data from unstructured documents is essential to bridge the gap between legacy documentation and modern automated configuration systems.

These challenges become more pronounced as device manufacturers adopt different documentation styles, operational constraints, and communication technologies. This necessitates that users interpret detailed technical specifications before achieving a correct configuration [9,10,11]. Consequently, even common setup tasks—such as selecting the appropriate wiring scheme, identifying supported voltage ranges, or choosing compatible communication modes—can become difficult for individuals without specialized technical expertise.

To assist users for this configuration process, AI-based setup assistance tools have emerged, providing step-by-step guidance through natural-language interactions. Although they are effective for familiar devices, current AI models rely solely on pre-trained knowledge and therefore fail to support newly released or previously unseen IoT devices [12]. As a result, users may receive incomplete or even misleading recommendations when the AI system encounters devices outside its encoded knowledge scope. This limitation underscores the importance of incorporating external technical documents into the configuration pipeline to maintain reliability as a new IoT hardware enters the market.

Existing datasheets exhibit heterogeneous layouts, complex tables, diagrams, and domain-specific terminology, making accurate extraction difficult [13]. Their formatting styles and parameter presentation methods vary widely across manufacturers, causing conventional rule-based approaches to produce fragmented or incorrect results.

While general-purpose multimodal document models excel at visual document understanding, they lack the domain-specific schema constraints required to normalize heterogeneous engineering units essential for safe IoT hardware configuration.

This diversity, combined with the lack of structured and normalized specifications, hinders effective integration of extracted information into downstream AI workflows. Even when textual content is available, the absence of a unified schema prevents direct alignment with device-configuration steps and limits its usefulness.

Although *Retrieval-Augmented Generation (RAG)* [14] offers a promising way to incorporate external knowledge, it depends on high-quality structured device information. Inadequate or inconsistent extraction degrades retrieval accuracy and restricts the ability of *RAG*-based systems to deliver precise, context-aware configuration guidance. Therefore, a robust method for converting raw datasheet content into consistent, machine-readable representations is essential for dependable AI-assisted IoT configuration.

In this paper, we propose a *generative AI-based technical data extraction tool* and show that it substantially improves a downstream *RAG*-based setup assistance tool for unseen IoT devices. Using a *schema-based extraction* strategy, the tool identifies key specifications—such as electrical characteristics, communication protocols, and operating conditions—from *PDF* or *HTML* documents and normalizes them into a unified structured format. This ensures consistent alignment of device information across different layouts and manufacturer styles while reducing ambiguity through standardized entries suitable for downstream processing.

In alignment with the goal of building a practical assistance tool for IoT application systems, this paper makes the following contributions:

- We design and implement a *generative AI-based technical data extraction tool* that converts heterogeneous *PDF/HTML* datasheets into a unified, schema-normalized representation with explicit provenance.
- We integrate the extracted structured knowledge into a local vector database to enable document-grounded retrieval for downstream *RAG*, thereby supporting newly released or previously unseen IoT devices without retraining the base *LLM*.
- We establish a manually verified golden QA benchmark and empirically show that higher-quality schema extraction yields a significant downstream gain in end-to-end configuration QA reliability compared with a commercial *RAG* baseline.

The extracted records are indexed in a local vector database using semantic embeddings, enabling document-grounded retrieval for downstream *RAG*-based setup assistance. To validate practical utility, we evaluate the schema-based extraction quality against manually verified ground truth and the downstream impact on end-to-end configuration QA reliability using a golden QA benchmark across multiple IoT datasheets.

These findings demonstrate the effectiveness of the proposed approach in strengthening the robustness and practicality of AI-assisted IoT configurations while providing a scalable foundation for future automated device-configuration support.

The remainder of this paper is organized as follows: Section 2 reviews related studies on AI-assisted IoT configuration, technical document understanding, and retrieval-augmented generation. Section 3 describes the challenges in supporting newly released or unseen IoT devices and motivates schema-normalized datasheet extraction. Section 4 presents the proposed technical data extraction tool, including the two-phase architecture, document preprocessing with visual fallback, schema design and normalization, vector database construction, and integration with the setup assistance workflow and user interface. Section 5 introduces the evaluation dataset and the manually verified golden QA benchmark, and defines the metrics and baseline protocol used for comparison. Section 5.4 reports the experimental results and provides a head-to-head comparison with the commercial baseline. Section 5.8 discusses the observed error patterns and operational implications, including document heterogeneity, evidence traceability, and the latency trade-off when visual fallback is triggered. Section 6 concludes the paper and outlines limitations and future work.

## 2. Related Works

In this section, we review studies related to AI-assisted IoT configuration, technical document understanding, structured knowledge modeling, and AI-based question answering. Although these works provide important foundations, to the best of our knowledge, we have not found a prior system that simultaneously integrates *LLM*-driven reasoning, configuration assistance, document grounding, schema-based extraction, and *QA* guidance into a unified IoT-support pipeline.

In [15], Dolan et al. extracted IoT device capabilities from vendor specifications using *NLP*-based analysis to identify the sensors, actuators, and functional properties of smart-home devices. Their method processes product webpages and technical documents to build structured device capability models. However, it does not incorporate *LLM* reasoning or interactive *QA* guidance.

In [16], Aguzzi et al. introduced an *LLM*-based macro-programming framework that transforms natural language descriptions of IoT system behavior into executable code. The method uses in-context learning to map user instructions into the aggregate computing paradigm for system-level modeling. However, it does not utilize technical documentation or provide question-answering functions to support configuration.

In [17], Zong et al. demonstrated the use of *GPT*-based *LLMs* to automate IoT tasks involving security analysis, dataset summarization, and programming support across multiple case studies. Their experiments show that fine-tuned *LLMs* can achieve high performance in various IoT automation tasks. However, the approach does not address structured schema extraction or interactive guidance grounded in technical documents.

In [18], Dong et al. proposed *ChatIoT*, an *LLM*-driven IoT security assistant using retrieval-augmented generation to support threat intelligence queries. The system constructs a unified knowledge base from heterogeneous IoT reports and datasets and improves reliability through template-based prompting. However, it focuses on security question-answering rather than IoT configuration or structured schema modeling.

In [19], Shastri et al. introduced *CollabIoT*, which converts high-level user intents into fine-grained access-control policies for transient IoT devices using an *LLM*. Their prototype demonstrates accurate policy generation and rapid device configuration. However, it does not utilize external documentation or offer an AI-based *QA* interface.

In [20], Zhang et al. developed a knowledge-graph question-answering method for IoT forensics, enabling investigators to retrieve forensic information through natural-language queries. Their solution supports semantic reasoning over IoT forensic data. However, it does not employ *LLM*-based reasoning or integrate technical documents such as manuals or datasheets.

In [21], Sobhan et al. proposed an *LLM*-assisted technical document *QA* system that combines vector retrieval with a structured-aware re-ranker to answer questions from equipment manuals. The approach handles tables and diagrams and achieves high faithfulness and relevancy in technical question-answering benchmarks. However, the system is specialized for document *QA* and does not support IoT configuration or device modeling tasks.

In [22], Gallo et al. presented a conversational agent that interprets natural-language user commands and generates automation rules for smart environments using *GPT-4*. It assists non-expert users in creating and modifying trigger-action rules on smart-home platforms. However, it does not incorporate technical documentation or schema-based modeling to guide configuration.

Table 1 summarizes the main research directions related to *LLM*-assisted reasoning, IoT configuration, document utilization, and structured extraction. Each line of work focuses on a specific aspect of IoT automation. In contrast, the proposed method integrates these capabilities within a unified framework to support device-configuration tasks using structured technical data. Based on this comparison, Section 3 summarizes the remaining challenges in supporting newly released or unseen IoT devices and introduces the motivations behind our proposed approach. In Table 1, a mark ∘ indicates that the corresponding capability is an explicit component of the method and is evaluated or demonstrated in the paper; × indicates that the capability is not addressed as a primary design objective.

## 3. Challenges and Motivation

In this section, we summarize the principal challenges encountered when extracting and utilizing technical information from IoT device documents and explains the motivation for an automated, scalable support mechanism.

### 3.1. Challenges

Despite the availability of general-purpose AI tools, building reliable IoT application systems remains difficult. The major obstacles supporting the motivation of our proposed tool are summarized in the following three categories.

#### 3.1.1. Complexity of Configuration and Device Selection for Non-Experts

For novice users without specialized engineering backgrounds, the initial phase of IoT system construction involves significant cognitive barriers, particularly in device selections and physical assemblies [23]. Non-experts often struggle to identify compatible sensors and edge devices, failing to distinguish between varying interface standards or voltage requirements [24]. Even when devices are correctly selected, the physical connection process—identifying pinouts, selecting pull-up resistors, and ensuring correct wiring, is prone to errors that can physically damage hardware [25]. General-purpose guides are often too generic, lacking specific, context-aware instructions needed to guide a beginner through the safe interconnection of a specific sensor model to a specific gateway [26].

#### 3.1.2. Inability of AI Models to Support Unseen Devices

While existing AI-based setup assistance tools can provide guidance for popular, well-documented hardware, they suffer from a critical “knowledge cutoff” [27]. Manufacturers frequently release new sensor variants or update firmware APIs, rendering the pre-trained knowledge of *Large Language Models (LLMs)* obsolete [28]. When users query an AI assistant about a newly released device that was not in its training set, the model often hallucinates, inventing plausible but incorrect pin configurations or driver commands. This unreliability forces users to manually verify AI suggestions against technical documents, negating the efficiency benefits of the AI assistant and potentially leading to deployment failures as highlighted in our abstract.

#### 3.1.3. Heterogeneity and Inconsistency in Technical Documentation

To ground AI responses in reality, systems must retrieve information from technical datasheets [29]. However, automating this extraction process is hindered by extreme heterogeneity in document formats and inconsistent technical representations [30]. Datasheets vary widely in layout, with critical parameters buried in complex tables, footnotes, or multi-column text blocks that confuse standard parsers. Furthermore, manufacturers use inconsistent terminology and diverse unit conventions [31,32]. This lack of structured, machine-readable specifications prevents standard *RAG* systems from effectively retrieving and normalizing the precise technical data required to generate accurate configuration support for users.

**Figure 2:**
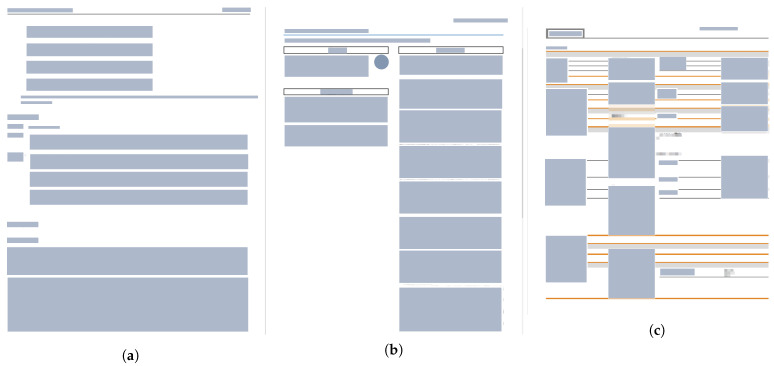
Visual categorization of layout heterogeneity in IoT datasheets. (**a**) Single-column; (**b**) Double-column; (**c**) Mixed-layout.

To illustrate the structural complexity encountered in real-world scenarios, Figure 2 presents a structural visualization of three representative datasheet layouts. While legacy documents often maintain a simple structure as abstracted in Figure 2a, modern specifications frequently adopt dense formats to maximize information density, as depicted in Figure 2b. The most significant challenge, however, arises from mixed layouts shown in Figure 2c, where manufacturers integrate sidebars, floating tables, and graphical elements in a non-linear arrangement. This extreme variability renders traditional rule-based parsers ineffective.

### 3.2. Motivation and Rationale of the Proposed Approach

Motivated by the heterogeneity and lack of machine-readable specifications described above, we propose an automated pipeline that combines layout-aware extraction with semantic normalization to enable scalable device support. The primary objective is to bridge the gap between unstructured technical documentation and the requirements of reliable AI-assisted configuration.

The proposed approach addresses the identified challenges through three specific mechanisms. First, to overcome layout variability and the limitations of rule-based parsing, a generative AI model is employed to perform schema-based extraction, converting diverse document structures into a unified format. Second, to resolve semantic inconsistencies, the proposal normalizes extracted fields, thereby ensuring data integrity for downstream logic. Third, to mitigate the latency in supporting new devices, the structured data is indexed in a vector database, allowing the setup assistance tool to retrieve up-to-date specifications via *RAG* without retraining the underlying model. This methodology aims to significantly reduce manual adaptation effort and improve the precision of configuration guidance, providing a scalable foundation for heterogeneous IoT environments.

## 4. Design of the Proposed Technical Data Extraction Tool

In this section, we present architectural principles and functional modules of the proposed generative AI-based technical data extraction tool. The system is engineered to support automated setups for IoT application systems by converting unstructured datasheet content into structured, queryable knowledge.

### 4.1. System Architecture

**Figure 3:**
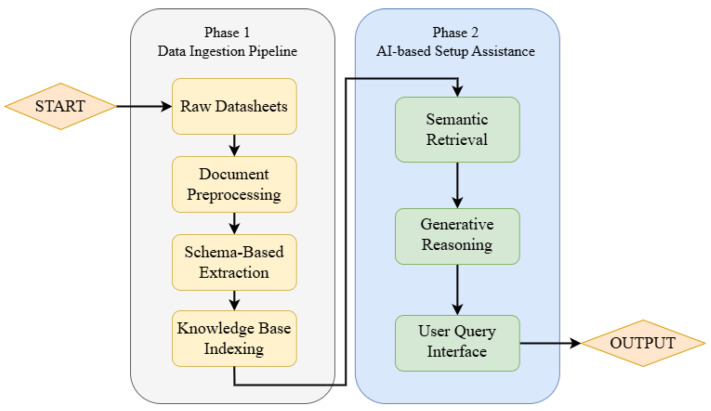
Two-phase architecture of proposed tool: datasheet ingestion/extraction/indexing (Phase 1) and document-grounded QA for configuration support (Phase 2).

The primary objective of the proposed tool is to extract structured key-value pairs from heterogeneous technical documents, thereby bridging the gap between the static, unstructured documentation and the dynamic configuration requirements of modern IoT environments. As illustrated in Figure 3, the overall architecture is structurally divided into two distinct operational phases: the *Data Ingestion Pipeline (Phase 1)* and the *AI-based Setup Assistance (Phase 2)*. This decoupled design ensures a clear separation of concerns between computationally intensive data preparation and real-time user interaction, allowing for efficient scalability and system maintenance.

*Phase 1* constitutes the offline construction of a reliable knowledge base, designed to transform raw information into machine-interpretable data. The process initiates with the ingestion of *Raw Datasheets* in diverse formats such as *PDF* or *HTML*. These documents typically contain noise and inconsistent layouts, necessitating a rigorous *Document Preprocessing* stage to clean, segment, and reconstruct the logical reading order. Following this, the core *Schema-based Extraction* module is employed to identify and normalize unstructured text into standardized semantic entities, ensuring that parameters like voltage or pin definitions are consistent across different manufacturers. The phase concludes with *Knowledge Base Indexing*, where the extracted structured data is embedded into high-dimensional vectors and stored in a database. This indexing step is critical for mitigating retrieval latency and enabling semantic similarity searches in the subsequent phase.

*Phase 2* executes the interaction workflow, enabling the system to function as an intelligent setup assistant. It begins at the *User Query Interface*, where the operator’s intent—ranging from simple parameter lookups to complex wiring scenarios—is captured in natural language. This input triggers the *Semantic Retrieval* engine, which queries the indexed knowledge base to fetch the most relevant technical context and schema-normalized facts. These retrieved segments are then fed into the *Generative Reasoning* module, where a *Large Language Model (LLM)* synthesizes the information to construct a grounded response. Finally, the system produces an actionable Output, providing the user with precise configuration guidance, wiring instructions, or compatibility warnings.

### 4.2. Document Preprocessing

In the *Document Preprocessing* stage, we first employ a mature open-source *PDF*-to-*Markdown* conversion tool (for *PDFs*) or *DOM* parsing (for *HTML*) to obtain a structured textual representation of each page. However, datasheet specifications are often embedded in rasterized content, such as mechanical drawings or pinout diagrams. To extract this information without relying on cloud-based APIs, the system integrates a locally deployed lightweight *Vision-Language Model (VLM)*. This module selectively performs *optical character recognition (OCR)* and scene understanding on image-based regions, converting visual schemas into textual descriptions that can be processed by the downstream extraction logic.

To suppress recurrent headers and footers that appear across pages [33], we adopt a majority-voting strategy. The global occurrence count c(x) of a segment *x* across all pages is calculated as follows:

(1) $$c\left(x\right)=\sum_{i=1}^{N}1\left(x\in S_{i}\right)$$

where *N* denotes the total number of pages, $S_{i}$ represents the candidate noise segments on page *i*, and 1(·) is the indicator function. Subsequently, the set of global noise segments $S^{*}$ is determined based on the occurrence ratio:

(2) $$S^{*}=\left\{x\;|\;\frac{c(x)}{N}\geq \theta \right\}$$

where θ∈(0,1] is a configurable threshold (typically set to 0.8).

To rigorously implement this noise removal process, we formalized the logic in Algorithm 1. This algorithmic approach ensures that device-specific metadata (appearing once) is preserved, while document-level metadata (recurrent artifacts) is effectively purged before embedding. Following this algorithmic filtering, a secondary cleaning phase further removes low-information elements, such as boilerplate legal disclaimers and invalid URLs. Fallback pagination is also employed to reconstruct logical boundaries when metadata is corrupted. By deferring complex inference, this stage ensures a clean, layout-aware corpus, thereby improving *RAG* recall and minimizing generative noise.

After preprocessing, the tool performs schema-based extraction to convert unstructured datasheet text into typed key-value records aligned with the predefined schema. Concretely, each document is segmented into overlapping chunks and processed independently by an *LLM* using a strict *JSON* output contract.

Since a specification may appear in multiple chunks, the tool aggregates chunk-level *JSON* outputs into a document-level record. For each schema field, we keep the normalized value, a confidence indicator derived from validation status, and provenance meta-data. When conflicting values are observed, we prioritize values with explicit numeric patterns and stronger local evidence and retain alternative candidates for manual inspection.

The *LLM* is instructed to output only *JSON* fields defined in the schema. If a field is not explicitly supported by evidence in the provided chunk, the tool requires the model to output null (rather than guessing). This design prevents speculative values from entering the knowledge base.
**Algorithm 1** Global header and footer removal**Require:** Pages: Set of document pages $\left\{p_{1},p_{2},\ldots ,p_{N}\right\}$**Require:** Threshold: Minimum occurrence ratio θ (e.g., 0.8)**Ensure:** CleanText: Document text with repetitive artifacts removed  1:GlobalCounts←∅  2:NoiseSet←∅  3:**for** p∈Pages **do**  4:      Segments←ExtractTopBottom(p)  5:      **for** s∈Segments **do**  6:            GlobalCounts[s]←GlobalCounts[s]+1  7:      **end for**  8:**end for**  9:Total←Length(Pages)10:**for** s∈Keys(GlobalCounts) **do**11:      **if** GlobalCounts[s]/Total≥Threshold **then**12:            Add *s* to NoiseSet13:      **end if**14:**end for**15:CleanText←∅16:**for** p∈Pages **do**17:      **for** block∈p **do**18:            **if** block∉NoiseSet **then**19:                 Append block to CleanText20:            **end if**21:      **end for**22:**end for**23:**return** CleanText

Each returned *JSON* is parsed and validated by a deterministic validator (type checks, unit patterns, and range constraints). If validation fails (e.g., malformed *JSON*, unit mismatch), the tool automatically re-prompts the *LLM* with the validation error summary and requests a corrected *JSON* output.

### 4.3. Schema Design

A standardized schema is essential to overcome the semantic inconsistencies inherent in multi-vendor datasheets. The schema defines a controlled vocabulary categorizing technical specifications into five key functional domains: device identification, communication parameters, sensor metrics, data acquisition characteristics, and environmental operating conditions.

To strictly enforce data consistency required for the vector database, we formalized the cleaning and normalization process into a deterministic algorithm. Unlike the probabilistic nature of *LLM* generation, this post-processing step ensures that all numerical values are converted to standard SI units (e.g., converting mV to V, μA to mA) and fall within physically plausible ranges.

Algorithm 2 details the procedural logic used to map raw LLM outputs to the standardized schema. The process iterates through each extracted field, verifies its data type, and applies a unit conversion factor if the detected unit differs from the base unit defined in the schema.
**Algorithm 2** Schema-guided unit normalization**Require:** RawExtracts: Key-value pairs from LLM (e.g., {voltage: “500 mV”})**Require:** Schema: Definitions of BaseUnit, Type, and Range**Require:** UnitMap: Conversion factors (e.g., $\left\{mV:10^{-3},k\Omega :10^{3}\right\}$)**Ensure:** CleanData: Normalized specifications  1:CleanData←∅  2:**for** (key,value)∈RawExtracts **do**  3:      **if** key∈Schema **then**  4:           **if** Schema[key].Type is **Numeric then**  5:                 (val,unit)←ParseValue(value)  6:                 base←Schema[key].BaseUnit  7:                 **if** unit≠base **and** unit∈UnitMap **then**  8:                       $val_{norm}\leftarrow val\times UnitMap\left[unit\to base\right]$  9:                 **else**10:                       $val_{norm}\leftarrow val$11:                 **end if**12:                 **if** $Schema\left[key\right].Min\leq val_{norm}\leq Schema\left[key\right].Max$ **then**13:                       Add $\left(key,val_{norm}\right)$ to CleanData14:                 **end if**15:            **else**16:                 Add (key,value) to CleanData17:            **end if**18:     **end if**19:**end for**20:**return** CleanData

As detailed in Table 2, the schema is implemented using a *JSON*-compatible structure that enforces data types and constraints for each field. Normalization rules are applied to map disparate terms to canonical field names and to convert values into standard SI units. To ensure long-term viability, the schema design incorporates an extensibility mechanism and a versioning policy. This allows the system to accommodate newly released devices with novel features without breaking backward compatibility for existing device models.

### 4.4. Vector Database Construction for RAG

To facilitate semantic grounding for the generation process, the preprocessed text segments are transformed into high-dimensional vector representations. An optimized embedding model is employed to encode both the semantic meaning and the structural context of each text chunk, allowing the proposal to represent heterogeneous technical descriptions within a unified embedding space.

A sliding-window chunking strategy with controlled overlap is adopted to preserve contextual continuity across segment boundaries, particularly for specifications that span multiple table rows or descriptive paragraphs. This design improves retrieval robustness by ensuring that semantically related parameters remain accessible as coherent units, even when distributed across complex document layouts. Specifically, we employed a chunk size of 512 tokens with a 128-token overlap. This configuration ensures that boundary-spanning specifications—such as a table header appearing on one page and its data on the next—are retained within the same semantic context window.

The core retrieval mechanism relies on cosine similarity to estimate the semantic proximity between a user’s natural-language query and the stored document vectors [34]. The similarity function is defined as:

(3) $$\mathrm{sim}\left(q,x_{i}\right)=\frac{q\cdot x_{i}}{∥q∥\;∥x_{i}∥}$$

where *q* represents the query vector and $x_{i}$ represents the embedding of the *i*-th document chunk. To retrieve the most pertinent context, the system performs a Top-*k* retrieval operation, defined as:

(4) $$R_{k}\left(q\right)={\mathrm{Top}}_{k}\left(\left\{\mathrm{sim}\left(q,x_{i}\right)|x_{i}\in X\right\}\right)$$

where *X* denotes the complete vector set. The value of *k* is tuned based on empirical validation to achieve a balance between coverage of important specifications and mitigation of irrelevant retrieval noise [35].

All embedding vectors, together with associated metadata such as source file, page index, and schema-mapped parameter type, are indexed using an approximate nearest-neighbor search structure. This enables efficient sub-linear retrieval even as the corpus expands with additional IoT devices and datasheets. As a result, the system avoids exhaustive scanning of entire manuals and instead restricts the reasoning scope of the *LLM* to only the most semantically grounded sections, significantly improving scalability and response determinism.

### 4.5. Integration with Setup Assistance Tool

The final component is the integration layer, which bridges the structured extraction pipeline with the interactive IoT setup assistant. This design decouples the AI backend from frontend logic, ensuring system-level flexibility and modularity.

**Figure 4:**
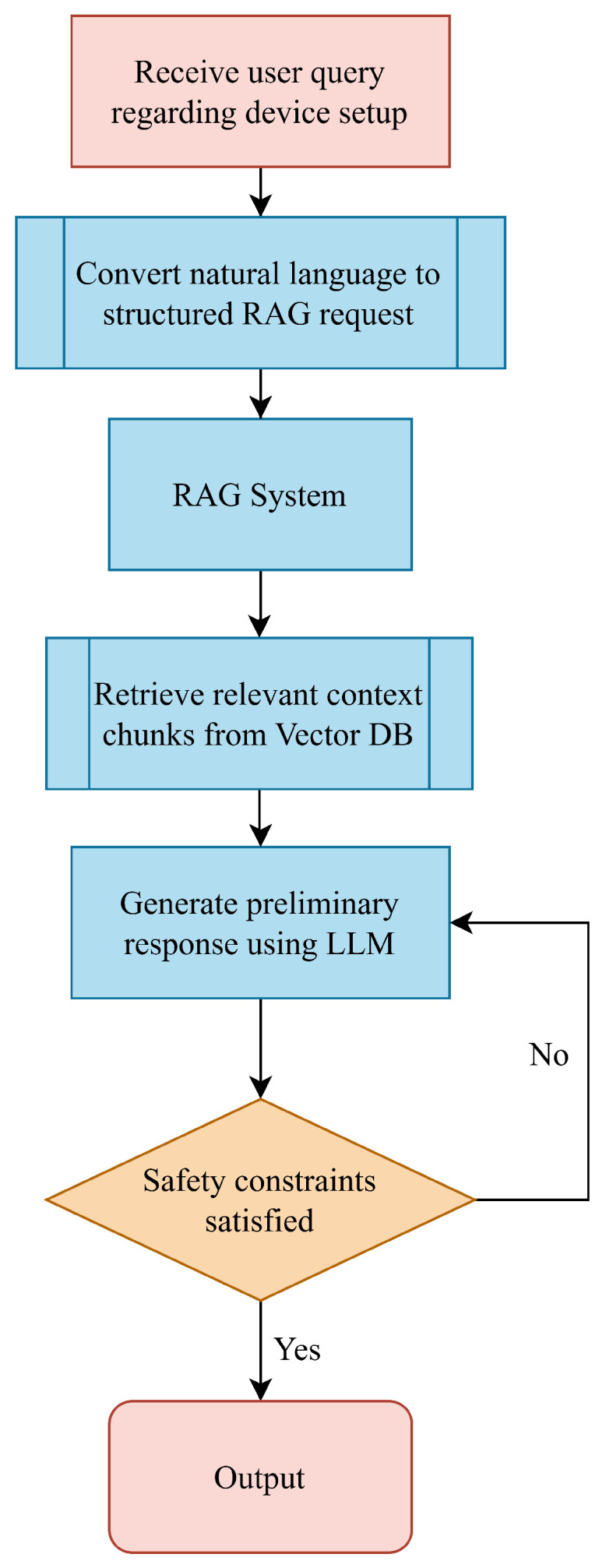
Runtime control flow of proposed tool-assisted setup guidance.

As illustrated in Figure 4, the operational workflow proceeds in four distinct stages:

1. **Query Parsing**: The assistant tool converts user configuration scenarios into structured requests forwarded to the *RAG* pipeline.
2. **Context Retrieval**: Relevant document segments are retrieved and fed into the *LLM* to generate grounded explanations.
3. **Safety Validation**: A deterministic validation layer screens the output against non-negotiable constraints.
4. **Response Generation**: If the output is verified, actionable guidance is returned; otherwise, the system suppresses speculative content and issues a fallback warning.

This structured integration guarantees that users receive guidance directly supported by authoritative evidences, significantly reducing the risk of hallucinated or unsafe operational steps.

### 4.6. User Interface

**Figure 5:**
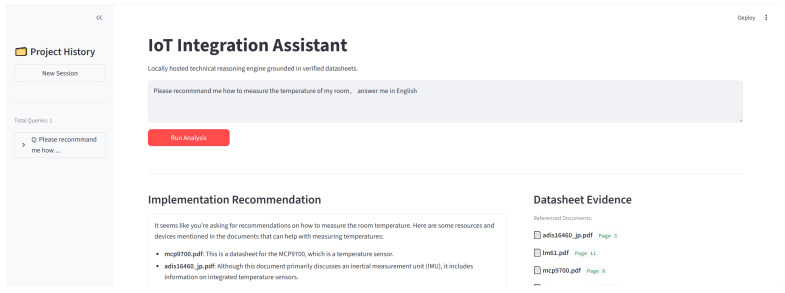
User interface of proposed tool.

The user interface serves as the primary interaction point between the operator and the proposed IoT configuration assistant, adopting a functional split-pane layout prioritized for transparency and evidence awareness as shown in Figure 5. The design features a project history sidebar on the left that functions as a persistent session log, recording chronological user queries to allow operators to navigate between distinct configuration contexts without losing track of prior steps. The central workspace handles query processing, where users submit natural-language requests and trigger the inference pipeline to receive actionable setup guidance synthesized in the implementation recommendation block. To support traceability and manual verification, the datasheet evidence panel on the right explicitly displays the provenance of the generated response by listing referenced document identifiers alongside their corresponding page numbers. By visually juxtaposing the generated recommendation with its supporting sources, the interface facilitates immediate grounding and allows users to trace extracted specifications back to the original datasheet context, thereby fostering trust calibration for safety-critical parameters such as voltage limits and interface compatibility.

Moreover, the central workspace is engineered to support iterative configuration workflows. Unlike static search engines, the system maintains a conversational state, allowing users to refine their queries based on previous outputs. For instance, if an initial wiring suggestion is incompatible with the user’s specific microcontroller, the user can provide feedback or additional constraints directly in the chat interface. The system leverages the context preserved in the session history to adjust its retrieval strategy and generate a corrected configuration plan, ensuring that the assistance adapts dynamically to the evolving requirements of the deployment scenario.

## 5. Evaluation Method

In this section, we present the evaluation method of the proposed generative AI-based technical data extraction tool. To validate practical utility, we consider the end-to-end *QA* correctness as a downstream task and analyze error sources from retrieval and extraction behaviors. Furthermore, we analyze the quality of the retrieved evidence to determine whether errors stem from missing/irrelevant context or from generation mistakes.

### 5.1. Experimental Setup and Evaluation Dataset

To evaluate the robustness and adaptability of the proposed extraction tool across heterogeneous technical documentation, we curated a diverse dataset comprising eight distinct datasheets from various IoT device manufacturers. The selection criteria prioritized variability in document structure, technical domain, and content density to ensure a rigorous assessment.

All experiments were conducted on a local workstation (CPU: Intel Core i7-12700F, GPU: RTX 5070 Ti, RAM: 32 GB). For generative reasoning, we utilized the locally deployed google/gemma-2-27b-it model. Semantic embeddings were generated using the text-embedding-3-small model with a chunk size of 512 tokens, indexed via FAISS. The average end-to-end processing time is on the order of 10 s per query, which increases when visual fallback is triggered. The visual fallback is triggered only when text-based extraction yields insufficient evidence (e.g., the target field is null after validation) or when the retriever returns no high-confidence context for the query.

#### 5.1.1. Document Selection and Characteristics

The dataset encompasses a broad spectrum of IoT components, ranging from environmental monitoring devices to industrial process control instruments. As detailed in Table 3, the documents exhibit significant structural variations:

- **Device Diversity:** The corpus covers varied signal types and operational complexities, including simple analog sensors (D-06, D-08), complex *6-DOF* inertial measurement units (D-03), and universal signal converters (D-02). This diversity tests the system’s ability to extract standardized schema fields from functionally distinct devices.
- **Layout Heterogeneity:** The dataset presents distinct formatting challenges. It includes concise single-column specifications (D-01, D-04), dense double-column technical manuals typical of complex integrated circuits (D-03, D-05), and mixed-layout documents (D-02) where standard text flows are disrupted by sidebars or embedded wiring diagrams.
- **Content Volume:** Document lengths range from brief 4-page summaries to comprehensive 26-page references, with a total corpus size of 121 pages. This variation evaluates the system’s capability to maintain context validity across both short, focused summaries and extensive, multi-page specifications.

#### 5.1.2. Ground Truth Construction

To quantitatively evaluate end-to-end *QA* correctness and downstream *RAG* performance, we established a “Golden” benchmark consisting of manually verified question-answer pairs. The benchmark was constructed through a rigorous semi-automated pipeline designed to ensure domain coverage and evaluate reasoning depth:

1. **Candidate Generation:** For each of the eight datasheets, an *LLM* was employed to scan the document and propose 150 candidate technical questions. To ensure comprehensive coverage of the IoT configuration lifecycle, the candidates were distributed across five distinct categories: *Device Identification*, *Communication & Setup*, *Input Specifications*, *Environmental & Mechanical*, and *Setup & Configuration*.
2. **Difficulty Stratification:** To assess the system’s capability beyond simple keyword matching, we categorized the *answerable* questions into three difficulty levels based on the complexity of retrieval and reasoning required:
  - **Easy (Explicit Retrieval):** The answer is explicitly stated in a single sentence or a simple list (e.g., “What is the model name?”). This tests basic retrieval accuracy.
  - **Medium (Structured Extraction):** The answer requires extracting values from complex tables or interpreting conditional parameters (e.g., “What is the current consumption at 5 V supply?”). This tests the system’s ability to handle structural heterogeneity.
  - **Hard (Multi-hop Reasoning):** The answer requires synthesizing information from multiple sections or understanding implicit constraints (e.g., “Which resistor value should be used for I2C pull-up at 100 kHz?”). This evaluates the system’s logical reasoning and context window retention.
3. **Human Verification and Refinement:** We reviewed the candidates and removed ambiguous items, corrected unsupported answers, and validated the category and difficulty labels for answerable questions. Each retained answer was required to be explicitly grounded in the datasheet text, tables, or figures. In addition, for unanswerable questions (negative controls), the experts verified that the queried information was not present anywhere in the corresponding datasheet.
4. **Final Compilation:** We selected 100 verified answerable *QA* pairs per document and additionally introduced 20 verified unanswerable questions per document (ground truth set to “N/A”). As a result, the final benchmark contains 120 samples per document, totaling 960 evaluation samples across eight datasheets (800 answerable + 160 unanswerable).

The distribution of these 960 questions across the defined technical categories is summarized in Table 4. This categorization ensures that the benchmark covers not only static specifications but also dynamic configuration parameters critical for real-world deployment, while the unanswerable subset provides a controlled way to evaluate over-answering behavior.

By incorporating difficulty stratification on the answerable subset, this benchmark allows for a fine-grained analysis of the system’s performance. While baseline models may perform adequately on “Easy” queries, the “Medium” and “Hard” categories are specifically designed to highlight the advantages of our proposed schema-based extraction and validation logic in handling complex, unstructured technical data. The additional unanswerable questions complement this analysis by measuring whether a system can appropriately abstain when the required evidence is absent.

#### 5.1.3. Information Extraction Using *ChatPDF*

This subsection describes the use of ChatPDF as a representative black-box *PDF QA* baseline. We selected *ChatPDF* as the primary baseline, because it represents a widely adopted, commercial-grade implementation of document *QA* using *OCR* and retrieval-augmented generation. Since *ChatPDF* encapsulates a strong “standard” *RAG* pipeline in practice, it serves as a rigorous reference point for evaluating whether our proposed extraction tool can improve document-grounded *QA* reliability on heterogeneous datasheets.

To minimize prompt-induced variance, we used a fixed instruction template for all queries (Listing 1). In *ChatPDF*, the uploaded *PDF* serves as the document context; therefore the placeholder {CONTENT} denotes the document text internally extracted by *ChatPDF* rather than any externally provided raw text.

For scoring, we mapped each question to its target schema field and compared the corresponding *JSON* value with the manually verified ground truth using the same evaluation rules as our tool. All baseline runs were completed within a single day under identical settings, and the same evaluation rules were applied to both systems.

**Table:** **Listing 1.** Prompt template in *ChatPDF* for each *QA* query.

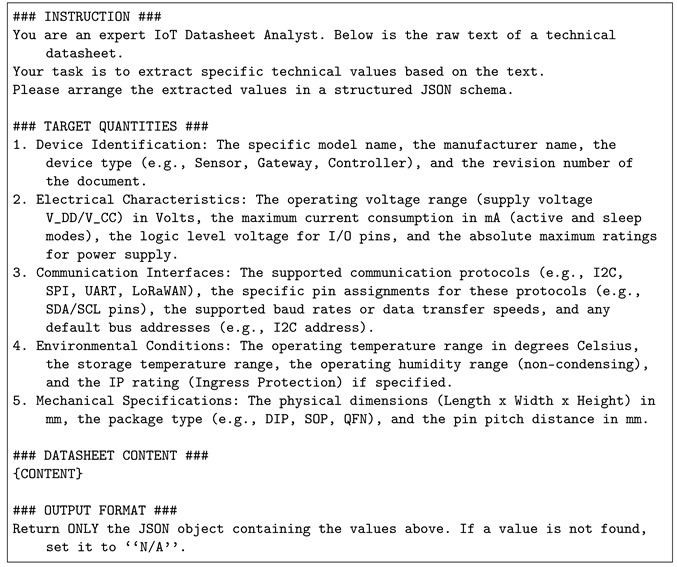

### 5.2. Evaluation Metrics

Consistent with the evaluation framework for information extraction, we score each question by a four-outcome rule: *correct answer (TP)*, *incorrect answer (FP)*, *omission (FN)*, or *correct abstention (TN)*. We define the confusion matrix components as follows:

- **True Positive (TP):** The system correctly extracts a value that matches the ground truth (e.g., Ground Truth: “3.3 V”, System: “3.3 V”).
- **False Positive (FP):** The system outputs an incorrect value for an answerable question, *or* outputs any concrete value when the ground truth is missing (i.e., over-answering on an unanswerable question).
- **False Negative (FN):** The system fails to extract a value (returns a missing indicator) when the ground truth contains a value.
- **True Negative (TN):** The system correctly reports a missing indicator when the information is indeed absent from the document (ground truth is missing).

In scoring, we treat “N/A” (*ChatPDF* output), “Not Available” (paper description), and *JSON* null (our tool output) as equivalent missing indicators. Any of these outputs is regarded as “missing”. Accordingly, for answerable questions a missing output is counted as an omission (FN), while for unanswerable questions a missing output is counted as a correct abstention (TN).

Based on these definitions, we utilize the following metrics to quantify performance:

(5) $$Accuracy=\frac{TP+TN}{TP+FP+FN+TN}$$

(6) $$Precision=\frac{TP}{TP+FP}$$

(7) $$Recall=\frac{TP}{TP+FN}$$

(8) $$Specificity=\frac{TN}{TN+FP}$$

(9) $$F1=\frac{2\cdot Precision\cdot Recall}{Precision+Recall}$$

*Precision* reflects the correctness among answered questions, while *Recall* reflects the rate of successfully recovering correct answers without omission, and F1 summarizes the trade-off between them. *Specificity* reflects the system’s ability to *abstain correctly* when the required information is absent, i.e., how often the model returns a missing indicator on unanswerable questions instead of fabricating a value. In the context of datasheet-grounded configuration support, high *Specificity* is essential to suppress hallucinated specifications and to prevent users from acting on nonexistent parameters.

For *Specificity*, TN and FP are computed only on the unanswerable subset (Ground Truth = “N/A”). In this subset, FP counts over-answering cases where the tool outputs any concrete value despite missing evidence.

At the same time, *Specificity* must be interpreted together with *Recall*: a conservative system can increase *Specificity* by frequently returning missing indicators, but this may also increase FN and reduce *Recall* on answerable questions. Therefore, we report both *Recall* and *Specificity* to characterize the trade-off between coverage (answering when evidence exists) and safety-oriented abstention (not answering without evidence).

### 5.3. Comparative Baseline

For evaluation each datasheet was uploaded in an isolated session (one session per document). Each *Golden* question was issued once in a single turn without follow-up prompts, and the first returned result was recorded.

For fairness and reproducibility, each datasheet was uploaded individually to *ChatPDF*, and each question was queried once using a fixed prompt template (Listing 1). We report head-to-head results between *ChatPDF* and our proposed generative AI-based technical data extraction tool.

### 5.4. Evaluation Results

In this section, we present the empirical findings of the experiments conducted on the curated dataset of heterogeneous IoT datasheets. To provide a rigorous diagnostic of behaviors, we analyze the performance of the baseline approach *ChatPDF* and the proposed generative AI-based technical data extraction tool separately, before synthesizing a comparative summary. The analysis focuses on the interplay between the retrieval completeness (*Recall*) and the generation precision, quantifying how the proposed multimodal architecture mitigates the limitations of traditional text-only extraction.

In addition to the original answerable benchmark, we extended the evaluation with deliberately unanswerable queries (*Ground Truth* = “N/A”) to measure whether a method can correctly abstain when the specification is absent. As a result, the full evaluation set contains 960 queries in total (800 answerable + 160 unanswerable across eight datasheets).

### 5.5. Performance Assessment of Baseline Approach

**Figure 6:**
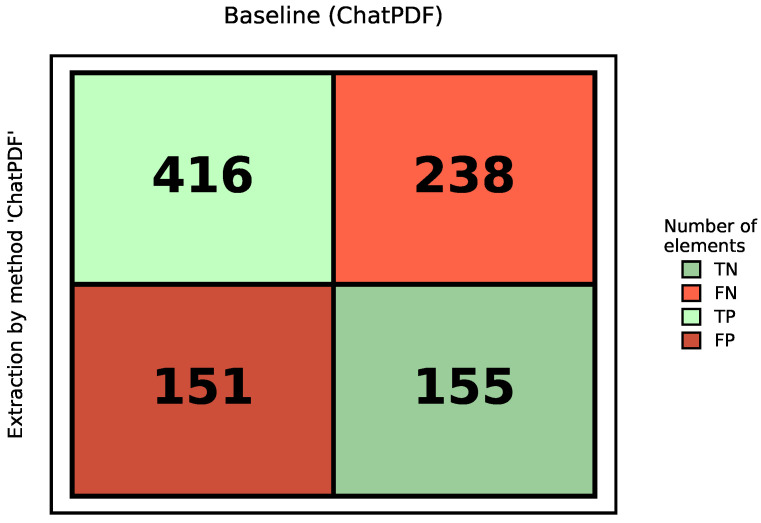
Confusion matrix for *ChatPDF*.

The baseline evaluation utilized the *ChatPDF* pipeline, which relies on standard *OCR* and text-based vector retrieval. Figure 6 visualizes the confusion matrix for the baseline method, categorizing the extraction outcomes into *True Positives (TP)*, *False Positives (FP)*, *False Negatives (FN)*, and *True Negatives (TN)*.

As illustrated in the matrix, the system correctly identified 416 items (*TP*, top-left) but failed to retrieve 238 existing specifications (*FN*, top-right). This significant red quadrant visually quantifies the high “Omission Error” rate, indicating that the baseline frequently returns “N/A” for valid questions. For the unanswerable subset, the baseline correctly abstained on 155 out of 160 queries as *TN*, but still produced 5 spurious values, which are counted as *FP*.

Table 5 details the derived performance metrics for the baseline. The dominant failure mode observed is **Omission Error**, represented by the high *FN* count (FN=238). This resulted in a *Recall* of 0.636, implying that nearly 37% of the critical technical specifications were missed. Meanwhile, the presence of unanswerable queries enables direct measurement of abstention behavior via *TN*.

**Impact of Document Heterogeneity on Baseline Failure:** A deeper investigation into the document-level performance reveals that the baseline’s reliability is highly correlated with document formatting. As illustrated by the specific case of Document D-01, which relies heavily on embedded raster images for dimension specifications, the baseline achieved a negligible extraction rate (*Accuracy* <7%). The text extraction layer failed to capture the callout values in the mechanical drawings, resulting in a complete loss of information. Similarly, in Document D-01, the mixed-layout formatting disrupted the logical reading order of the standard vector retrieval, causing the *LLM* to miss key parameter associations. These results underscore that while standard *RAG* pipelines perform adequately on “clean” text, they lack the robustness required for legacy or visually complex datasheets.

### 5.6. Performance Assessment of Proposed Tool

**Figure 7:**
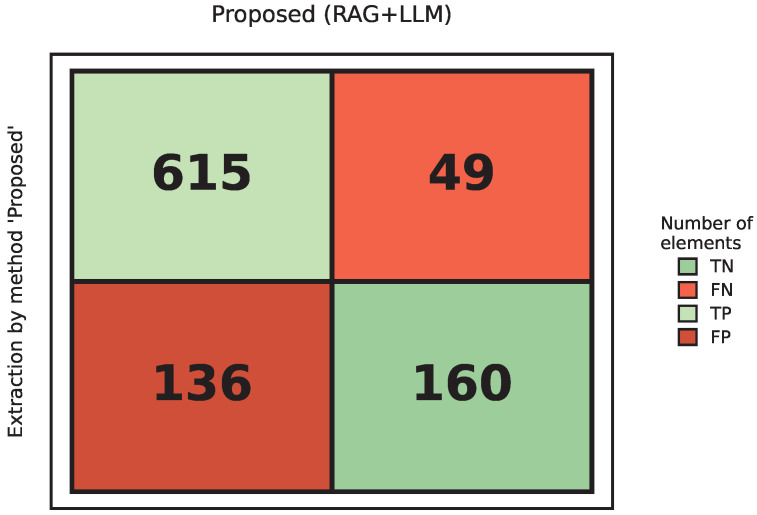
Confusion matrix for proposed tool.

The proposed method introduces a schema-guided extraction strategy augmented by a *Visual Fallback* mechanism. Figure 7 presents the confusion matrix for the proposed method. A visual comparison with the baseline matrix highlights a dramatic topological shift in error distribution: the dense block of *FN* has been effectively migrated to *TP*, while the method also maintains reliable abstention on unanswerable queries.

Table 6 presents the specific performance indicators for our tool. The most significant achievement is the elevation of *Recall* to 0.926, reducing the absolute number of missed questions from 238 to just 49. This improvement validates the efficacy of the *Adaptive Multimodal Fallback* logic: when text retrieval yields low confidence, the system leverages the vision-language model to read visual elements such as pinout diagrams and mechanical drawings. In addition, the unanswerable subset confirms conservative behavior: the proposed method returns “N/A” for all 160 negative queries (*TN* = 160), i.e., no spurious-value outputs are observed for missing specifications.

Overall, the proposed pipeline substantially reduces omission errors on image-heavy or mixed-layout pages, while keeping the incorrect-answer rate controlled.

### 5.7. Comparative Evaluation and Net Improvement

To succinctly quantify the advancements achieved by the proposed tool, we conduct a direct head-to-head comparison of the aggregate metrics and a stratified analysis of performance across different question complexities.

#### 5.7.1. Aggregate Performance Gains

Table 7 provides the overall metric comparison across the full dataset of 960 queries (including unanswerable *TN* queries). The empirical data demonstrates a clear superiority of the hybrid approach over the unimodal baseline. The **Net Improvement** row highlights the system’s impact: an increase of 199 correctly answered questions (+47.8% relative improvement in *TP*), a reduction of 189 missed questions (−79.4% reduction in *FN*), and improved abstention behavior (*TN* +5), while also reducing *FP*.

#### 5.7.2. Performance Stratification by Query Complexity

A critical dimension of our evaluation is assessing how the system copes with increasing cognitive load. As defined in Section 5, the test questions were stratified into “Easy” (Explicit Retrieval), “Medium” (Structured Extraction), and “Hard” (Multi-hop Reasoning). Table 8 breaks down the F1-score performance across these categories. Note that this difficulty-based analysis is computed on the answerable subset, since unanswerable queries are labeled “N/A” by design and are not associated with difficulty levels.

The data reveals a widening performance gap as complexity increases:

1. **Easy Queries:** For simple metadata extraction (e.g., “Model Name”), both systems perform adequately, though the proposed method achieves near-perfect reliability (F1=0.94). This confirms that for basic text retrieval, standard *RAG* is often sufficient.
2. **Medium Complexity:** When extracting values from dense tables (e.g., “Current consumption at 5 V”), the baseline performance drops to 0.61. The proposed method, aided by the schema-guided retrieval which effectively “flattens” tabular structures for the *LLM*, maintains a high score of 0.88.
3. **Hard Reasoning:** The most striking difference is observed in multi-hop reasoning tasks (e.g., checking implicit constraints or converting units). The baseline degrades significantly (F1=0.45), often failing to connect disparate pieces of information. In contrast, the proposed method demonstrates robust reasoning capabilities (F1=0.79), representing a 75.5% relative gain. This resilience is mainly attributed to evidence-grounded retrieval combined with schema-guided normalization and post-validation, which reduces unsupported guesses and improves consistency on multi-step queries.

In summary, while the baseline struggles to maintain coherence on complex tasks, the proposed generative AI-based technical data extraction tool delivers consistent, high-fidelity extraction across the full spectrum of document difficulties, and also exhibits reliable abstention behavior on unanswerable queries, establishing a stronger benchmark for automated technical data extraction.

### 5.8. Discussion

The experimental results presented in Section 5.4 provide evidence that addressing document heterogeneity is the pivotal challenge in automated IoT data extraction. The proposed tool, by integrating schema-guided retrieval with a visual fallback mechanism, successfully mitigates the limitations inherent in traditional unimodal (text-only) *RAG* systems.

#### 5.8.1. Interpretation of Findings

The most significant finding is the dramatic improvement in **Recall** (from 0.636 to 0.926) and **Overall Accuracy** (from 0.595 to 0.807) on the full evaluation set that includes unanswerable queries. This quantitative leap validates our core hypothesis: technical datasheets are multimodal entities where critical information is often encoded in non-textual formats (e.g., mechanical drawings, timing diagrams).

- **Visual Semantics as Safety Net:** The case study of Document D-01 demonstrates that when *OCR* fails, visual reasoning is not merely an enhancement but a necessity. The system’s ability to “see” the mounting hole dimensions directly from the schematic diagram transformed a near-zero extraction rate into a usable output.
- **Reliable Abstention on Missing Specifications:** Introducing unanswerable queries enables explicit measurement of whether a method refrains from guessing when information is absent. The proposed tool correctly returned “N/A” for all 160 unanswerable queries (*TN* = 160), whereas the baseline produced a small number of spurious values (5 cases). This behavior is essential for configuration safety, where guessing absent limits can be more harmful than returning “N/A”.
- **Reasoning over Retrieval:** The superior performance on “Hard” complexity questions (F1=0.79) indicates that the system moves beyond keyword matching. The gain on hard queries suggests that the pipeline benefits from structured prompting and validation, which encourages unit/range checks and cross-section consistency when synthesizing retrieved evidence.

#### 5.8.2. Trade-Offs and Operational Implications

Compared with the baseline, the proposed method achieves a large reduction in *FN* (238 → 49) while keeping *FP* controlled (151 → 136 on the full set). In an industrial “Human-in-the-loop” (HITL) workflow, this trade-off is operationally advantageous. A *FN* (missing value) imposes a high cognitive load on the engineer to manually search the document, whereas an incorrect value can often be detected quickly when evidence and provenance are provided. Therefore, the proposed tool significantly reduces the total engineering time required to digitize legacy specifications.

Another critical operational implication concerns the traceability and verification of the extracted data. Unlike standard “black-box” *QA* systems, our proposed tool explicitly links every generated specification to its source page in the datasheet, as displayed in the user interface. This source attribution feature allows operators to instantly cross-reference the AI’s output with the original document, ensuring the authenticity of critical parameters before physical implementation. In a practical setup scenario, this ability to validate data sources is far more valuable than raw retrieval speed, as it directly mitigates the risk of hardware damage caused by blind reliance on AI.

#### 5.8.3. Comparison with Standardized Approaches

Although standardized technologies, such as device descriptors or *XML*-based schemas used in mobile and smart home domains, are offering deterministic parsing with minimal computational overhead, they fundamentally rely on the availability of pre-defined files. In the context of generic IoT components, such files are unfortunately often absent. Therefore, our AI-based extraction tool can be viewed as an upstream generator, not a competitor to standardized technologies. By converting unstructured legacy PDFs into normalized data, our tool effectively produces the structured inputs required by standardized technologies, thereby enabling their applications to previously unsupported hardware.

We acknowledge that parsing a structured file like *XML* is orders of magnitude faster and consumes less memory than processing a PDF via a *Vision-Language Model*. However, this comparison is only valid when the structured file pre-exists. For generic IoT components lacking such files, the practical alternative is manual data entry, which is time-consuming and error-prone. From this perspective, although our method requires GPU resources, it provides a critical automation capability that standard parsers cannot offer, yielding significantly higher end-to-end efficiency compared to manual digitization.

Recent standards such as the *Model Context Protocol (MCP)* require structured context to connect *LLMs* with data sources. Our proposed extraction tool aligns with this standard by serving as an upstream data provider. By transforming raw PDF datasheets into structured schemas, our tool generates the necessary context that *MCP*-compliant servers require, effectively extending the reach of *MCP* to cover offline or legacy technical documentation.

## 6. Conclusions

### 6.1. Summary

This paper addressed the practical difficulty of using heterogeneous datasheets to support reliable IoT system configuration. A generative AI-based technical data extraction tool was developed, converting *PDF/HTML* datasheets into schema-normalized records with explicit provenance and validation, and we assessed its downstream utility in a document-grounded setup QA workflow.

Experiments on eight datasheets with 121 pages and 960 manually verified *QA* pairs with 800 answerable + 160 unanswerable ones demonstrated that the proposed pipeline substantially improves end-to-end reliability compared with a commercial *RAG* baseline (*ChatPDF*), especially on visually complex or mixed-layout documents. In particular, the proposed method markedly reduces omission errors by recovering specifications that are difficult to access via text-only parsing, while also exhibiting reliable abstention behavior on missing specifications through correct “N/A” outputs for unanswerable queries.

The research questions raised in the Introduction are answered as follows:

- **RQ1: Handling heterogeneity.** The two-phase pipeline combines layout-aware preprocessing with a visual fallback path, enabling robust extraction across single-/double-column and mixed-layout datasheets.
- **RQ2: Extraction reliability.** Using the golden benchmark with added unanswerable queries, the proposed approach achieves an aggregate F1-score of 0.869, improves *Recall* and *Overall Accuracy* over the baseline, and maintains correct abstention on missing specifications (*TN* = 160), indicating more complete and dependable recovery of technical specifications.
- **RQ3: Reasoning with constraints.** For hard questions requiring cross-section synthesis (e.g., constraint checking and parameter disambiguation), schema-guided prompting and deterministic validation reduce unsupported guesses and help produce configuration-relevant answers grounded in evidence.

### 6.2. Key Benefits

The proposed tool offers three practical benefits for IoT configuration support:

- **Reliable and schema-consistent specification extraction.** By applying schema-based extraction with deterministic validation, the tool converts heterogeneous *PDF/HTML* datasheets into a unified set of key specifications (e.g., voltage ranges, interfaces, and operating constraints). This reduces format-induced omissions and improves the completeness and stability of downstream configuration support across vendors and document styles.
- **Evidence traceability for transparent verification.** Each extracted record and *QA* response is accompanied by explicit provenance (document identifier and page-level references), enabling users to trace critical values back to the original datasheet context. This evidence-first design supports trust calibration and practical safety checks when handling failure-sensitive parameters such as electrical limits and interface compatibility.
- **Sustained support for unseen devices via local retrieval grounding.** The extracted structured knowledge is indexed in a local vector database and retrieved by semantic similarity to ground *RAG*-based answering. As a result, the setup assistance workflow can provide consistent guidance for newly released or previously unseen IoT devices without retraining the underlying *LLM*, yielding substantially higher end-to-end *QA* reliability compared with a commercial *RAG* baseline.
- **Simplified operation for non-expert users.** By automating the complex extraction of technical parameters, our tool shifts the burden of verifying electrical and wiring constraints from the user to AI. This significantly simplifies the operational procedure for non-expert users, allowing them to focus on high-level application logic rather than low-level hardware debugging.

### 6.3. Key Limitations

Several limitations remain and motivate further improvements:

- **Parameter ambiguity.** The proposed tool can confuse semantically adjacent fields (e.g., absolute maximum vs. recommended operating conditions), which contributes to residual false positives when section boundaries are unclear.
- **Runtime latency and efficiency.** Although the extraction latency of about 10 s for each query precludes use in real-time control loops, it is well within the acceptable tolerance for *configuration tasks*. Since datasheets are typically processed once during the initial system setup, the emphasis is placed on the extraction accuracy over millisecond-level speed.
- **Schema portability.** The present schema is optimized for IoT sensors. Extending coverage to other device classes requires domain-specific schema engineering. However, the underlying pipeline—including the layout-aware preprocessing, visual fallback, and validation logic—remains generic and reusable across different hardware domains.

### 6.4. Future Work

Future work will prioritize practical accuracy gains and better runtime efficiency.

First, to mitigate residual False Positives caused by semantic ambiguity, we will implement a contrastive validation layer. This module will explicitly check for adjacent numeric columns in the source table and apply heuristic constraints—such as ensuring that ’Maximum’ values strictly exceed ’Operating’ values—before finalizing the extraction.

Second, we will improve throughput by reducing the frequency and cost of visual fallback through caching, early-exit policies, and lightweight diagram-region selection, so that only evidence-critical regions are processed.

Third, we will expand the schema and evaluation to cover broader device categories and refine the benchmark with more configuration-oriented multi-hop questions, while also expanding the coverage of unanswerable queries to further stress-test conservative “N/A” behavior in real deployment workflows. 

## Figures and Tables

**Table 1:** Comparison of key characteristics between related works and our proposal.

Characteristic	[15]	[16]	[17]	[18]	[19]	[20]	[21]	[22]	Proposed
Technical document utilization	∘	×	×	∘	×	×	∘	×	∘
IoT configuration/automation support	∘	∘	∘	×	∘	∘	×	∘	∘
Structured schema extraction	∘	×	×	×	∘	∘	∘	×	∘
LLM-assisted reasoning	×	∘	∘	∘	∘	×	×	∘	∘
AI-based QA or guidance	×	×	×	∘	×	∘	∘	∘	∘

**Table 2:** Representative fields in normalized schema for configuration-oriented specification extraction.

Category	Field Name	Semantic Constraint/Type
Device Identification	device_name	String (e.g., “DHT11 Sensor”)
	model_number	String (e.g., “DHT11”)
	manufacturer	String (optional)
Electrical & Limits	supply_voltage_range	Numeric range in V (e.g., 3.0–5.5)
	logic_level_voltage	Numeric in V (optional)
	abs_max_voltage	Numeric in V (optional, disambiguated)
	current_active	Numeric in mA (optional)
	current_sleep	Numeric in μA/mA (optional)
	power_constraints	Structured text/list (optional)
Interface & Setup	protocols	Set {I2C, SPI, UART, 1-Wire, …}
	i2c_address	Hex/integer (optional)
	pin_mapping	Dict/list (e.g., SDA/SCL, MISO/MOSI/SCK)
	port	GPIO name/number (optional)
	baud_rate	Numeric in bps (UART, optional)
	recommended_wiring	Text/list (optional)
Environmental	operating_temperature	Numeric range in °C
	storage_temperature	Numeric range in °C (optional)
	humidity_range	Percentage range (optional)
Mechanical	dimensions	Numeric tuple in mm (optional)
	package_type	String (optional)

**Table 3:** Detailed specifications of selected IoT datasheets.

Doc ID	Device Category	Device Type	Pages	Layout Format
D-01	Sensor	Current Sensor	4	Single-column
D-02	Signal Conditioner	Universal Signal Converter/	8	Mixed-layout
		Trip Amplifier		
D-03	Sensor	6-DOF Inertial Sensor	26	Double-column
D-04	Sensor	Dust Sensor	5	Single-column
D-05	Sensor	Analog Temperature Sensor	25	Double-column
D-06	Sensor	Analog Temperature Sensor	18	Double-column
D-07	Sensor	pH/ORP Sensor	24	Mixed-layout
D-08	Sensor	Angle Sensor	11	Single-column
Total	–	–	121	–

**Table 4:** Distribution of 960 “Golden” *QA* pairs across technical categories.

Technical Category	Count per Doc	Total Questions
Device Identification	14	112
Input Specifications	24	192
Communication & Setup	29	232
Environmental & Mechanical	19	152
Setup & Configuration	34	272
Total	120	960

**Table 5:** Detailed performance metrics for *ChatPDF*.

Metric Class	Count/Value	Description
True Positives (TP)	416	Correctly extracted values matching ground truth.
False Positives (FP)	151	Incorrect values or spurious outputs when the correct answer is “N/A”.
False Negatives (FN)	238	Missed answers (returned “N/A”) for answerable questions.
True Negatives (TN)	155	Correctly returned “N/A” for unanswerable questions.
**Overall Accuracy**	0.595	Ratio of correct outputs over all queries (including TN).
**Precision**	0.734	Probability that an extracted value is correct.
**Recall**	0.636	Probability that an existing value is found.
**Specificity (N/A subset)**	0.969	$TN/(TN+FP_{\mathrm{NA}})$ on unanswerable questions only; here $FP_{\mathrm{NA}}=5$.
**F1-Score**	0.681	Harmonic mean of Precision and Recall.

**Table 6:** Detailed performance metrics for proposed tool.

Metric Class	Count/Value	Description
True Positives (TP)	615	Significant increase due to visual fallback.
False Positives (FP)	136	Incorrect values; controlled despite higher recall.
False Negatives (FN)	49	Drastic reduction in missed information.
True Negatives (TN)	160	Correctly returned “N/A” for all unanswerable queries.
**Overall Accuracy**	0.807	Ratio of correct outputs over all queries (including TN).
**Precision**	0.819	Improved by schema constraints and deterministic validation.
**Recall**	0.926	Near-complete coverage of answerable specifications.
**Specificity (N/A subset)**	1.000	$TN/(TN+FP_{\mathrm{NA}})$ on unanswerable questions only; here $FP_{\mathrm{NA}}=0$.
**F1-Score**	0.869	Balanced high performance.

**Table 7:** Aggregate performance comparison across full dataset.

Method	TP	FP	FN	TN	Overall Accuracy	F1-Score	Recall
Baseline (ChatPDF)	416	151	238	155	0.595	0.681	0.636
Proposed	615	136	49	160	0.807	0.869	0.926
Net Improvement	+199	−15	−189	+5	+0.212	+0.188	+0.290

**Table 8:** Performance gap analysis with F1-score.

Difficulty Level	Baseline F1	Proposed F1	Relative Gain
Easy (Explicit Retrieval)	0.78	0.94	+20.5%
Medium (Structured Table)	0.61	0.88	+44.2%
Hard (Multi-hop Reasoning)	0.45	0.79	+75.5%

## Data Availability

Data are contained within the article.
